# Surgical anatomy of hypoglossal canal for various skull base surgeries

**DOI:** 10.1007/s00276-023-03126-7

**Published:** 2023-03-17

**Authors:** Sneha Guruprasad Kalthur, Supriya Padmashali, Chachuu Bhattarai, Chandni Gupta

**Affiliations:** 1 Department of Anatomy, Kasturba Medical College, Manipal, Manipal Academy of Higher Education, Manipal, 576104 India; 2 Department of Anatomy, Rajarajeshwari Medical College and Hospital, Bengaluru, 560074 India; 3 Department of Anatomy, Manipal College of Medical Sciences, Pokra, Nepal

**Keywords:** Hypoglossal canal, Skulls, Neurosurgeons, Radiologist, Anatomical landmarks

## Abstract

**Purpose:**

Anatomical knowledge of the hypoglossal canal is very important in relation to drilling of occipital condyle, jugular tubercle etc. So, this study was conducted to identify various morphometric and morphological features of the hypoglossal canal and its distance from adjacent structures relative to stable and reliable anatomic landmarks.

**Methods:**

The study was performed on 142 hypoglossal canals of 71 adult human dry skulls. The parameters measured were the transverse, vertical diameter, depth of the hypoglossal canal. The distances from the hypoglossal canal to the foramen magnum, occipital condyle and jugular foramen were also noted. In addition, the different locations of the hypoglossal canal orifices in relation to the occipital condyle were assessed. The different shapes and types of the hypoglossal canal were also noted.

**Results:**

There was significant difference (*p* < 0.05) in measurements taken on the right and left sides in males and females. The intracranial orifice of hypoglossal canal was present in middle 1/3rd in 100% of occipital condyle for both genders. The extracranial orifice of the hypoglossal canal was found to be in the anterior 1/3rd in 99% and 93.7% for male and female, respectively. Simple hypoglossal canal with no traces of partition was found to be more in males and females. The most common shape noted was oval both in males and females (71.8% and 68.7% respectively).

**Conclusion:**

The results of the dimensions of the hypoglossal canal and its distance from other bony landmarks will be helpful for neurosurgeons to plan which surgical approaches should be undertaken while doing various surgeries in posterior cranial fossa.

## Introduction

Hypoglossal Canal (Anterior Condylar Canal) is a pair of bony canal situated very close to occipital condyles and jugular foramen. It extends from jugular process of occipital bone to the basiocciput. The canals are directed laterally and slightly forward and, transmits hypoglossal nerve, meningeal branch of ascending pharyngeal artery and an emissary vein [16]. Occasionally, the canal is divided by a spicule of bone leading to variation as dual hypoglossal canal. Variations with respect to race and gender has been reported earlier [1].

Hypoglossal canal can be involved in various pathological conditions requiring surgical interventions. There are many surgical approaches for various surgeries which requires proper morphometry of hypoglossal canal. Extreme lateral approach for vertebral artery aneurysms, meningiomas, chondrosarcoma. Lateral combined approach for tumors of clivus and combine approach for glomus jugulare tumors. Posterolateral approach to foramen magnum, transcondylar, supracondylar and paracondylar approaches for lower clivus, craniovertebral junction, hypoglossal canal and mastoid foramen respectively. Far lateral, dorsolateral, suboccipital approaches for lower clivus and craniovertebral junction, lateral approach for petroclival region [3, 6, 9, 14, 15]. However, transcondylar approach is preferred. During transcondylar surgical approach, occipital condyle is drilled from posterior aspect which threatens the opening of hypoglossal canal [6, 7]. Also, utmost care should be taken in case of atlanto-occipital dislocation where screw fixation is required from the articular mass of C1 to the occipital condyle [4]. In all these surgical approaches hypoglossal nerve is at risk. Anatomical knowledge of the hypoglossal canal is important during drilling of occipital condyle, jugular tubercle and lateral mass of C1 also. Considering its importance in planning surgical approaches the present study was carried out to determine the detailed morphometry and morphology of hypoglossal canal (HC).

## Materials and methods

The study was conducted on 142 hypoglossal canals of 71 adult human dry skulls, obtained from department of Anatomy. Among which 110 were of male and 32 were of female. The skulls with any damage in posterior cranial fossa, which would affect the study, were excluded from the study. Sliding Vernier Calipers and divider and scale were used to take morphometric measurements.

**Fig. 1:**
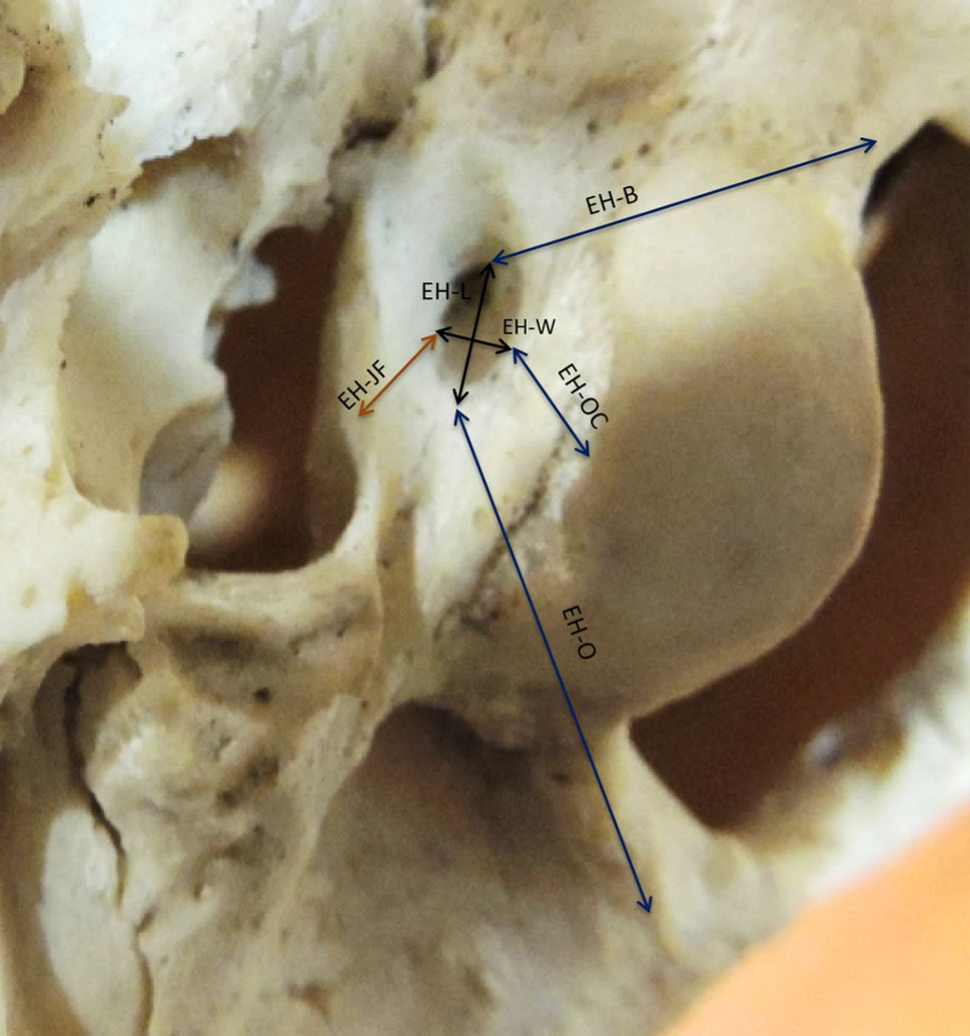
Showing anterolateral view of base of skull. The metric parameters taken for Extra cranial hypoglossal canal (EH

A. Metric parameters for Extra cranial hypoglossal canal (EH) taken were: (Fig. 1).

1. Vertical diameter (EH-V).
2. Transverse diameter (EH-T).
3. Distance between EH to occipital condyle (EH-OC).
4. Distances from the EH to the jugular foramen (EH-J).
5. Distances from the EH to the basion (EH-B).
6. Distances from the EH to the opisthion (EH-O).

**Fig. 2:**
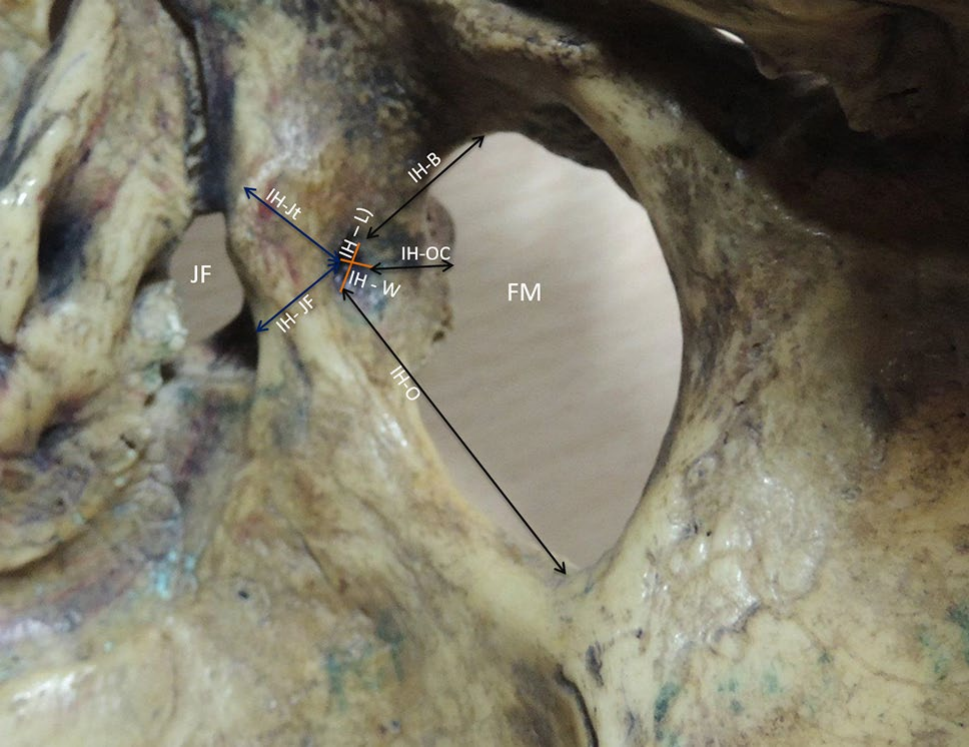
Showing posterolateral view of cranial cavity. The metric parameters taken for Intra cranial hypoglossal canal (IH), FM- Foramen magnum

B. Metric parameters for Intracranial Hypoglossal canal (IH) taken were: (Fig. 2).

1. Vertical diameter (IH-V).
2. Transverse diameter (IH-T).
3. Distance between IH to occipital condyle (IH-OC).
4. Distances from the IH to the jugular foramen (IH-J).
5. Distance between IH to jugular tubercle (IH-Jt).
6. Distances from the IH to the basion (IH-B).
7. Distances from the IH to the opisthion (IH-O).
8. Hypoglossal canal depth i.e. from the intracranial part of the canal to the extracranial part (IH-EH).

**Fig. 3:**
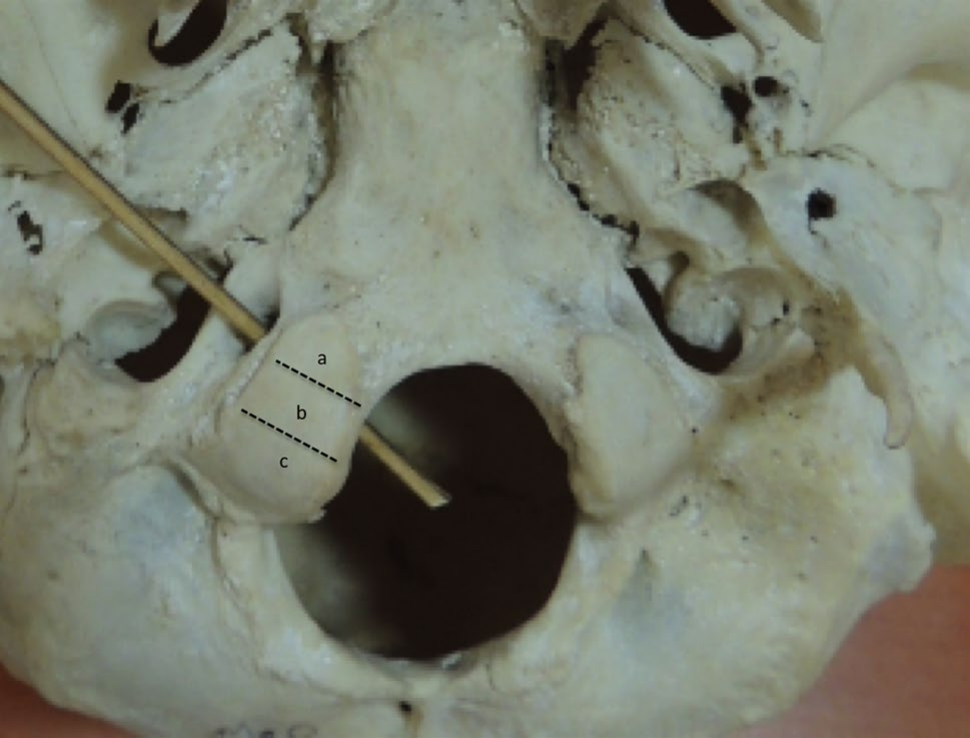
Showing top view of base of skull. The location of hypoglossal canal in relation to occipital condyle

C. Position of hypoglossal canal in relation to occipital condyle– maximum AP axis of condyle was measured and divided into 3 equivalent parts and classified as location a for anterior 1/3rd, location b for middle 1/3rd, location c for posterior 1/3rd (Fig. 3).

## Non morphometric parameters noted were

A. Shape of hypoglossal canal as round or oval.

**Fig. 4:**
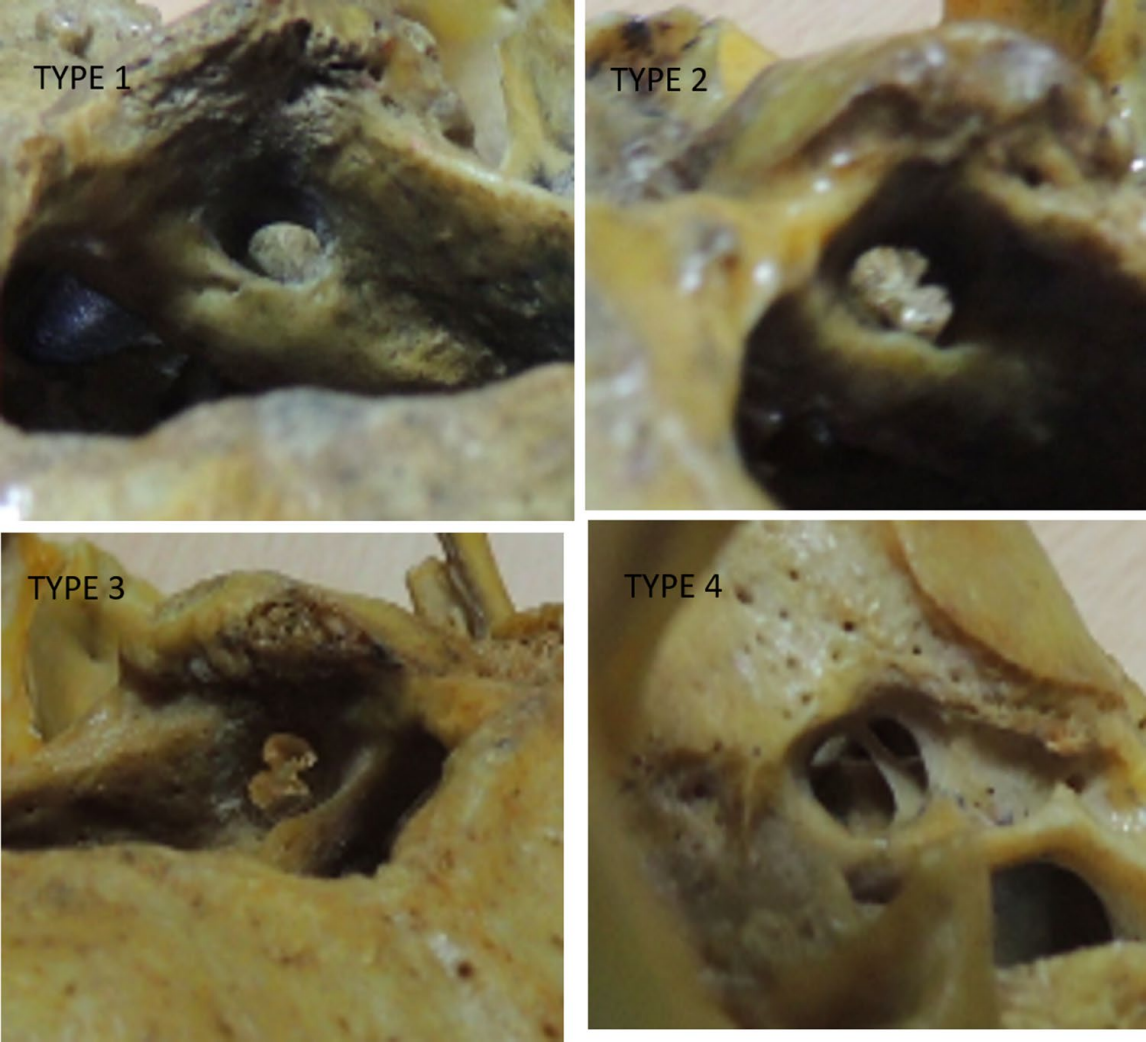
Showing anterolateral view of skull. Various types of hypoglossal canal according to osseous spur. Type1- Simple Canal, Type2- One osseous spur at margin either inner or outer aspect, Type3- two osseous spur anywhere in the canal, Type-4-Complete division either inner or outer margin

B. Types of hypoglossal canal–classified into 5 types according to Hauser G and De Stefano GF [5] (Fig. 4).

1. Type 1-No traces of partition, i.e. the presence of simple canal.
2. Type 2-Traces of partition, i.e. one bony spur (spine) present either at the margin of the inner or outer opening of the canal or inside it.
3. Type 3-Traces of partition, i.e. two or more bony spurs (spines) present anywhere along the canal.
4. Type 4-Complete bony hypoglossal bridging present either in internal or external part of the canal.
5. Type5-Complete bony hypoglossal bridging extending along the whole canal.

The morphometric and non-morphometric data were tabulated and segregated according to sex and skull side. Descriptive statistics (mean, standard deviation) were evaluated for all the parameters collected from the skull measurements. For all the analyses, *p* < 0.05 was accepted as statistically significant and statistical analysis was performed using IBM SPSS Statistics for Windows Version 20.0 (USA).

## Results

Measurements were taken from 71 dry skulls of known sex, 55 males (77.4%) and 16 females (22.5%). The results obtained from the metric parameters are presented in Tables 1 and 2.

**Table 1:** Metric parameters of extracranial hypoglossal canal (EH) cm

Parameters	Male (*n* = 55)			Female (*n* = 16)			Total		
	Right (Mean ± SD)	Left (Mean ± SD)	*P* value	Right (Mean ± SD)	Left (Mean ± SD)	*P* value	Male (*n* = 110) (Mean ± SD)	Female (*n* = 32) (Mean ± SD)	*P* value
EH-V	0.66 ± 0.09	0.71 ± 0.13	0.01*	0.63 ± 0.10	0.68 ± 0.15	0.24	0.69 ± 0.12	0.65 ± 0.13	0.44
EH-T	0.50 ± 0.71	0.49 ± 0.09	0.78	0.46 ± 0.10	0.49 ± 0.07	0.38	0.49 ± 0.08	0.48 ± 0.09	0.26
EH-OC	0.81 ± 0.19	0.77 ± 0.18	0.19	0.73 ± 0.19	0.64 ± 0.19	0.18	0.79 ± 0.19	0.68 ± 0.19	0.64
EH-J	0.38 ± 0.07	0.40 ± 0.12	0.49	0.39 ± 0.07	0.48 ± 0.19	0.07	0.39 ± 0.10	0.44 ± 0.15	0.04*
EH-B	1.79 ± 0.18	1.77 ± 0.17	0.59	1.66 ± 0.16	1.74 ± 0.22	0.09	1.77 ± 0.18	1.70 ± 0.20	0.75
EH-O	4.05 ± 0.25	4.09 ± 0.25	0.13	4.09 ± 0.35	4.14 ± 0.36	0.34	4.07 ± 0.25	4.11 ± 0.36	0.0000*

*p < 0.05- significant

**Table 2:** Metric parameters of intracranial hypoglossal canal (IH)

Parameters	Male			Female			Total		
	Right	Left	*P* value	Right	Left	*P* value	Male	Female	*P* value
IH-V	0.64 ± 0.10	0.68 ± 0.11	0.01*	0.66 ± 0.11	0.74 ± 0.10	0.04*	0.66 ± 0.11	0.70 ± 0.11	0.44
IH-T	0.45 ± 0.08	0.47 ± 0.09	0.24	0.45 ± 0.10	0.44 ± 0.09	0.79	0.57 ± 0.15	0.45 ± 0.09	0.93
IH-OC	0.93 ± 0.19	0.89 ± 0.17	0.14	0.80 ± 0.16	0.83 ± 0.09	0.45	0.91 ± 0.18	0.81 ± 0.13	0.09
IH-J	0.54 ± 0.47	0.55 ± 0.49	0.65	0.75 ± 0.41	0.78 ± 0.41	0.41	0.55 ± 0.48	0.77 ± 0.40	0.0000*
IH-Jt	0.78 ± 0.18	0.77 ± 0.14	0.81	0.86 ± 0.22	0.76 ± 0.17	0.02*	0.78 ± 0.16	0.81 ± 0.20	0.08
IH-B	1.63 ± 0.15	1.61 ± 0.15	0.38	1.59 ± 0.15	1.55 ± 0.15	0.31	1.62 ± 0.15	1.57 ± 0.15	0.72
IH-O	3.07 ± 0.28	3.07 ± 0.26	1.0	3.03 ± 0.23	3.13 ± 0.27	0.03*	3.07 ± 0.27	3.08 ± 0.25	0.95
IH-EH	0.90 ± 0.16	0.89 ± 0.20	0.73	0.90 ± 0.18	0.78 ± 0.19	0.09	0.90 ± 0.18	0.84 ± 0.19	0.57

*p < 0.05-significant

The vertical diameter of hypoglossal canal both extracranial and intracranial were significantly different between right and left side of male skull (*p* < 0.05), while in female skulls there was significant difference between right and left side in parameters like IH-V, IH-O and IH-Jt (*p* < 0.05). Similarly, when data was compared in male and female skulls parameters like EH-J, EH-O, IH-J showed significant difference (*p* < 0.05).

**Fig. 5:**
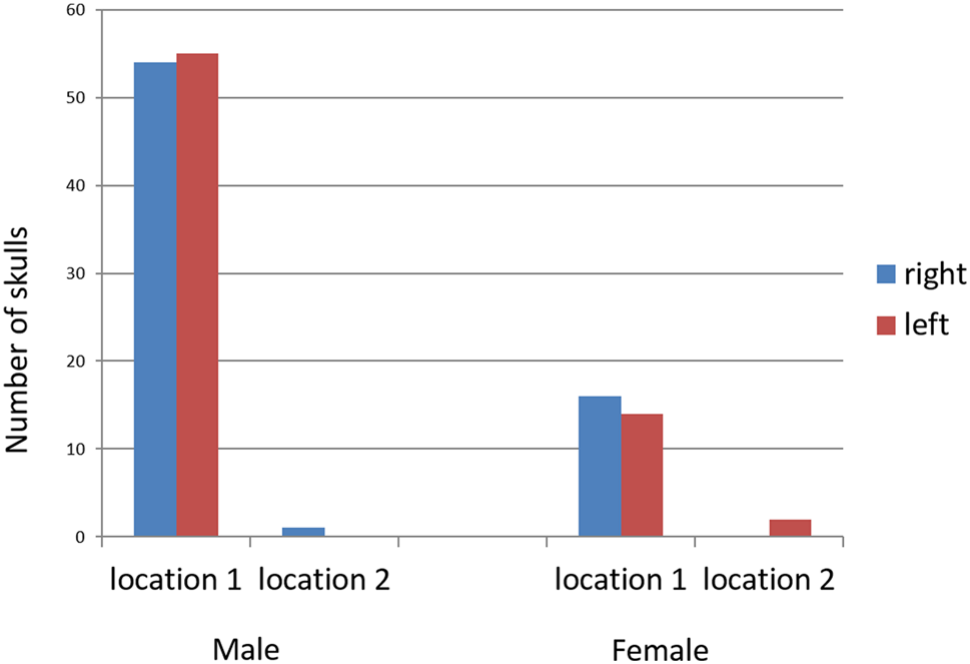
Graph showing the right and left side location of HC in relation to occipital condyle in both sexes

The locations of the hypoglossal canal in relation to the occipital condyle are represented in Table 3. It was found that the intracranial opening of hypoglossal canal was present in location 2 (middle 1/3^rd^) in 100% of occipital condyle for both genders. The extracranial opening of the hypoglossal canal was found to be in the location 1 (anterior 1/3^rd^) in 99% and 93.7% for male and female respectively. When the right and left side location of HC were analyzed separately, the frequency of variation in the location was almost similar (Fig. 5).

**Table 3:** Locations of intracranial and extracranial orifices of hypoglossal canal relative to the occipital condyle expressed in percentage

	Total	
	Male (*n* = 110)	Female (*n* = 62)
Location of Extra cranial Hypoglossal Canal		
Location 1 (Anterior 1/3rd)	99.09	93.75
Location 2 (Middle 1/3rd)	0.91	6.25
Location 3 (Posterior 1/3rd)	0	0

	Total	
	Male (*n* = 110)	Female (*n* = 32)
Location of Intra cranial Hypoglossal Canal		
Location 1 (Anterior 1/3rd)	0	0
Location 2 (Middle 1/3rd)	100	100
Location 3 (Posterior 1/3rd)	0	0

The HC was also classified into types. Type I was found to be more in males and females followed by type II, than type IV. Type III was least to be seen (Table 4). When the right and left side typing was analyzed separately, the frequency of variation was dissimilar. The most common shape noted was oval both in males and females (71.8% and 68.7% respectively) (Table 5). It was also noted that oval shape was dominant on left side as compared to right in both the gender.

**Table 4:** Various Types of Hypoglossal Canal expressed in percentage in male and female skull

Type	Male		Female		Total	
	Right (*n* = 55)	Left (*n* = 55)	Right (*n* = 16)	Left (*n* = 16)	Male (*n* = 110)	Female (*n* = 32)
Type I	83.6	40	62.5	25	43.6	43.7
Type II	32.7	40	25	37.5	36.3	31.2
Type III	5.4	1.8	6.2	12.5	3.6	9.3
Type IV	14.5	18.1	6.2	25	16.3	15.6

**Table 5:** Various shapes of extracranial hypoglossal canal expressed in percentage

Shape	Male		Female		Total	
	Right (*n* = 55)	Left (*n *= 55)	Right (*n* = 16)	Left (*n* = 16)	Male (*n* = 110)	Female (*n* = 32)
Round	32.8	23.7	37.5	25	28.2	31.3
Oval	67.2	76.3	62.5	75	71.8	68.7

## Discussion

To reduce the morbidity and mortality in craniovertebral surgeries the thorough knowledge of morphometry of base of skull is inevitable. The specific knowledge of morphometry of hypoglossal canal is pivotal to reduce the damage of hypoglossal nerve and also the related surrounding cranial nerves, great vessels in several craniovertebral surgeries.

Parvindokht et al. conducted a morphometric analysis of hypoglossal canal. They found that extracranial and intracranial transverse diameter of hypoglossal canal as 0.31 cm and 0.29 cm. Distance of hypoglossal canal to opisthion, basion and jugular tubercle as 3.38, 1.25 and 1.05 cm [13]. Ogut E et al. conducted a study on 18 human fixed cadaver heads bilaterally. They measured the diameter of internal opening of HC, the distance of intracranial openings of HC from the jugular foramen and jugular tubercle. They found that intracranial transverse diameter of hypoglossal canal was 0.22 cm. Distance of hypoglossal canal to jugular foramen and jugular tubercle as 1.08 and 0.87 cm [12]. In our study intracranial transverse diameter of hypoglossal canal was 0.50 cm. Distance of hypoglossal canal to jugular foramen and jugular tubercle were 0.66 and 0.79 cm. These differences in results might be racial differences as they have conducted the study in Iranian population and our study was done in Indian people.

Nikumbh et al. conducted a morphological analysis of hypoglossal canal. They found that extracranial AP and transverse diameter of hypoglossal canal in male, female as 0.63, 0.59 and 0.53, 0.51 cm [11]. These results were almost similar to our study as we got them as 0.69, 0.65 cm and 0.49, 0.48 cm in males, females respectively.

During transcondylar resection posterior one-third of occipital condyle is resected. Therefore, the distance between between OC and HC is important. In present study distance between extracranial orifice to OC ranged from 0.6 to 0.9 cm with an average of 0.79 cm in males and in females the range varied from 0.4 to 0.87 cm with an average of 0.68 cm. Similarly distance from intracranial orifice to occipital condyle range varied to be as 0.73 to 1.09 cm with average of 0.91 cm and in female average as 0.81 cm with range of 0.68 to 0.94 cm. This is nearly similar to that reported by Wen et al., the mean distance between the posterior aspect of the occipital condyle and the intracranial opening of hypoglossal canal is 0.84 cm (range 0.6–1 cm) [17]. In study conducted by Fetouh et al. the averaged distance ranged from 0.4 to 0.7 cm from intracranial orifice and Naderi et al. found the value as 0.4–0.7 cm [2, 10]. Muthukumar et al. found that the distance from the intracranial opening of hypoglossal canal to the posterior aspect of the occipital condyle as 1.2 cm [8]

From our study it was observed that the opening of extracranial hypoglossal canal was located close to anterior 1/3^rd^ of occipital condyle and intracranial hypoglossal canal orifice was located close to middle 1/3rd of occipital condyle, which was in agreement with the results got from Naderi et al. and Wen et al. [10, 17]. So, the exact position of the intracranial and extracranial opening of hypoglossal canal is vital during condylectomy. Too posteriorly placed intracranial opening of hypoglossal canal may create complication and prevent the transcondylar approach.

The exact osseous characteristics in relations with the specific canal types with neighboring structures was noted. The hypoglossal canal can be divided by an osseous bridge (type IV) that separates the meningeal branch of ascending pharyngeal artery from the hypoglossal nerve. In our study, this was present in more number on the left side (25% of the skulls in female and 18% in males) as compared to right side (6.2% in female and 14.5% in males). Nikumbh et al. in their study found both side complete bony septum in 3% of skulls. All skulls were male skulls those who have both side complete bony septum. They also found the bony spicule in 25% of skulls [11]. In our study we found (type II) one osseous spur (spine) in 36.3% in males and 31.2% in females.

The study of hypoglossal canal and its variant is essential not only to anatomists but also to forensic experts, neurosurgeons and anthropologists. As we know any skull base surgeries requires proper planning before intervening, as many neurovascular structures traverse close by. The dimensions of the hypoglossal canal and its relative relation to other bony landmarks are helpful for radiologists and neurosurgeons while intervening with cases like schwannoma of hypoglossal nerve in posterior cranial fossa. Knowledge of type and locations of extracranial and intracranial orifices of hypoglossal canal are of significance while performing procedures like condylectomy and limit the transcondylar approach.

## Conclusion

The study was conducted on 142 hypoglossal canals of 71 adult human dry skulls. Various parameters of hypoglossal canal and its relative distance from different anatomical landmarks was measured. In addition, the locations of the hypoglossal canal orifices in relation to the occipital condyle were assessed. The different shapes and types of the hypoglossal canal were also noted. It was seen that there was significant difference in measurements taken on the right and left sides in males and females. The intracranial orifice of hypoglossal canal was present in middle 1/3rd in relation to occipital condyle in all cases for both genders. The extracranial orifice of the hypoglossal canal was found to be in the anterior 1/3rd in 99% males and in 93.7% for female. Type I HC was found to be more in both sexes. The most common shape noted was oval both in males and females. Neurosurgeons requires proper planning before doing any surgeries in areas near skull base, as many neurovascular structures traverse at this region. The results of the dimensions of the hypoglossal canal and its distance from other bony landmarks will be helpful for neurosurgeons to plan which surgical approaches should be undertaken while doing various surgeries in posterior cranial fossa.

## Data Availability

Not applicable.
